# Differential Dynamics of Transposable Elements during Long-Term Diploidization of *Nicotiana* Section *Repandae* (Solanaceae) Allopolyploid Genomes

**DOI:** 10.1371/journal.pone.0050352

**Published:** 2012-11-21

**Authors:** Christian Parisod, Corinne Mhiri, K. Yoong Lim, James J. Clarkson, Mark W. Chase, Andrew R. Leitch, Marie-Angèle Grandbastien

**Affiliations:** 1 Institut Jean-Pierre Bourgin, UMR 1318 INRA-AgroParisTech, INRA-Versailles, Versailles, France; 2 School of Biological Sciences, Queen Mary University of London, London, United Kingdom; 3 Jodrell Laboratory, Royal Botanic Gardens, Kew, Richmond, Surrey, United Kingdom; University of Gottingen, Germany

## Abstract

Evidence accumulated over the last decade has shown that allopolyploid genomes may undergo drastic reorganization. However, timing and mechanisms of structural diploidization over evolutionary timescales are still poorly known. As transposable elements (TEs) represent major and labile components of plant genomes, they likely play a pivotal role in fuelling genome changes leading to long-term diploidization. Here, we exploit the 4.5 MY old allopolyploid *Nicotiana* section *Repandae* to investigate the impact of TEs on the evolutionary dynamics of genomes. Sequence-specific amplified polymorphisms (SSAP) on seven TEs with expected contrasted dynamics were used to survey genome-wide TE insertion polymorphisms. Comparisons of TE insertions in the four allopolyploid species and descendents of the diploid species most closely related to their actual progenitors revealed that the polyploids showed considerable departure from predicted additivity of the diploids. Large numbers of new SSAP bands were observed in polyploids for two TEs, but restructuring for most TE families involved substantial loss of fragments relative to the genome of the diploid representing the paternal progenitor, which could be due to changes in allopolyploids, diploid progenitor lineages or both. The majority of non-additive bands were shared by all polyploid species, suggesting that significant restructuring occurred early after the allopolyploid event that gave rise to their common ancestor. Furthermore, several gains and losses of SSAP fragments were restricted to *N. repanda*, suggesting a unique evolutionary trajectory. This pattern of diploidization in TE genome fractions supports the hypothesis that TEs are central to long-term genome turnover and depends on both TE and the polyploid lineage considered.

## Introduction

Recent investigations of allopolyploid taxa (i.e. species originating from hybridization of divergent genomes associated with doubling of chromosomes) have revealed that their genomes are dynamic, undergoing drastic structural and functional alterations referred to as diploidization [1], [2], [3], [4], [5]. Such genome differentiation seems to go along with the stabilization of nascent polyploids, but our understanding of these processes occurring over thousands/millions of years is still far from comprehensive [6], [7], [8], [9], [10].

Diploidization involves intra- and intergenomic rearrangements, including elimination of DNA sequences and amplification or reduction of repetitive sequences. Genomic reorganization could decrease pairing between homeologous chromosome and/or promote reproductive isolation between nascent polyploid lineages and their progenitors [7], [11], [12], [13], [14], [15]. Accordingly, hybridization has been highlighted as ‘genomic shock’, inducing rapid genomic reorganization [14], [16], [17].

A substantial fraction of plant genomes is made of transposable elements (TEs), thus mechanisms triggering long-term diploidization are likely to involve such repetitive sequences [18], [19], [20], [21], [22], [23]. Comparative genomics between descendants of progenitor diploids and polyploids is scarce, but revealed that TE genomic fractions undergo considerable changes subsequent to polyploidy (reviewed in [14]). In addition to transpositional activity, TEs can be substrates for unequal or illegitimate recombination, potentially resulting in substantial chromosomal repatterning [24], [25], [26], [27].

*Nicotiana* (Solanaceae) is a promising genus to explore the long-term impact of allopolyploidy on genome dynamics [28], [29], [30]. Several studies of *Nicotiana* species have produced a well-dated phylogenetic tree revealing several allopolyploid groups of different ages [31], [32], [33] and highlighted the impact of repetitive sequences on long-term diploidization [34]. Allopolyploid *N. tabacum* (up to 200 KY old) and synthetic allopolyploids showed restructuring of TE fractions, with massive losses of some TE sequences [13], [34], [35], [36]. Older *Nicotiana* allopolyploids have revealed considerable exchange of repetitive sequences among subgenomes [34], [37] and offered convincing evidence that a nearly complete structural differentiation of homeologous genomes occurred in less than 5 MY. However, to what extent various TE sequences participated in this high genomic turnover remains an open question. *Nicotiana* section *Repandae* (four extant species derived from a common allopolyploid ancestor) offers the opportunity to understand the molecular mechanisms underlying long-term diploidization. This clade originated 4.5 MY ago from hybridization between ancestors of extant species of *N*. sections *Sylvestres* and *Trigonophyllae* as maternal and paternal progenitors, respectively [32], [37]. The allopolyploid section currently includes *N. nudicaulis, N. repanda, N. stocktonii* and *N. nesophila*, and their evolutionary relationships are well known. *Nicotiana nudicaulis* is sister to, and morphologically distinct from, the three remaining species; it diverged 2–3 MY ago, whereas *N. stocktonii* and *N. nesophila* diverged from *N. repanda* more recently (1 MY ago). These allopolyploid genomes showed considerable sequence turnover as well as substantial change in genome size [34], [37], [38].

Here, we investigated dynamics of seven TEs: two short interspersed nuclear elements (SINEs: Au and TS), two miniature inverted-repeat transposable elements (*Stowavay* MITEs: Ns1 and Nt2) and three long terminal repeats (LTR) retrotransposons (i.e. the *copia*-like Tnt1 and Tnt2, and the short terminal repeat retrotransposon in miniature, TRIM). These TEs are known to display contrasting evolutionary dynamics in *N. tabacum* and its diploid progenitors *N. sylvestris* and *N. tomentosiformis*, providing useful evidence about their overall dynamics in genus *Nicotiana*. Tnt2 is a young, active element, abundant and highly conserved in *N. tabacum* (Deloger and Grandbastien, unpublished data). Tnt2 is 2- to 3-fold more abundant in *N. sylvestris* than in *N. tomentosiformis*, with few shared insertions and much amplified numbers in response to allopolyploidy [36]. To contrast with Tnt2, we selected an older, less abundant Tnt1 population [39], displaying many common insertions shared by *N. sylvestris* and *N. tomentosiformis* and limited proliferation in response to allopolyploidy [13], [36]. TRIM was selected because it is an ancient family conserved among monocotyledonous and dicotyledonous plants [40], and is heterogeneous in *N. tabacum* (Deloger and Grandbastien, unpublished data), indicating low levels of recent amplification. The two SINE families also show contrasting evolutionary patterns. TS elements are young composite SINEs that recently increased in the *N. tabacum* lineage [41], [42], whereas Au elements are ancient SINEs conserved among monocotyledonous and dicotyledonous plants [43]. They are less conserved, albeit more abundant, than TS elements in *N. tabacum* [41]. Finally, we included two MITEs, Ns1 and Nt2, for which only sequence data are available [44], as representative of DNA transposons.

This set of contrasting TE populations maximizes the chances of assessing differential TE dynamics during long-term polyploid evolution. We investigated their patterns of insertion polymorphisms within the well-defined phylogenetic framework offered by *Nicotiana* section *Repandae*, using a genome-wide sequence-specific amplified polymorphism (SSAP) approach [45]. Our aims were to assess (i) to what extent TE dynamics participated in the long-term genome turnover of polyploid *Nicotiana* and (ii) whether different TE types presented specific evolutionary trajectories after polyploidization. We show that restructuring of TE genomic fractions in allopolyploids is dependent on both the polyploid species and the TE, with some TE populations showing evidence of proliferation in specific polyploid species. This pattern supports the high levels of genome turnover reported from cytogenetics approaches [34] and indicates that TE genome fractions have been highly reorganized during long-term diploidization.

## Materials and Methods

### Plant Material

*Nicotiana* accessions were collected from various germplasm collections (Table 1). We selected four accessions of *N. sylvestris* (section *Sylvestres*), the only species in the section and the most closely related to the maternal progenitor of section *Repandae,* and six accessions of *N. obtusifolia*, formerly known as *N. trigonophylla*, (section *Trigonophyllae*), a species most closely related to the diploid paternal progenitor. Fifteen accessions of allopolyploid section *Repandae* represented the four currently recognized species: *N. nudicaulis* (five accessions), *N. repanda* (six accessions), *N. stocktonii* (two accessions) and *N. nesophila* (two accessions). Sequencing of the polymorphic plastid *trnS-G* locus further confirmed the taxonomic status of most accessions (data not shown).

**Table 1:** *Nicotiana* accessions investigated in this study.

Section	Species	Accession name	Abbreviation	Source
*Sylvestres* (2n = 2x = 24)	*Nicotiana sylvestris*	A047503026	syl1	Nijmegen Botanical Garden (NL) b,c
	*Nicotiana sylvestris*	TW137	syl2	USDA (US) d
	*Nicotiana sylvestris*	ITB626	syl3	Tobacco Institute of Bergerac (F) e
	*Nicotiana sylvestris*	934750319	syl4	Nijmegen Botanical Garden (NL) b
*Trigonophyllae* (2n = 2x = 24)	*Nicotiana obtusifolia* a	ITB614	tri1	Tobacco Institute of Bergerac (F) e,f
	*Nicotiana obtusifolia* a	TW98	tri2	USDA (US) d
	*Nicotiana obtusifolia*	TW143	tri3	USDA (US) d
	*Nicotiana obtusifolia*	ITB627	tri4	Tobacco Institute of Bergerac (F) e,f
	*Nicotiana obtusifolia*	ITB518	tri5	Tobacco Institute of Bergerac (F) e
	*Nicotiana obtusifolia*	894750176	tri6	Nijmegen Botanical Garden (NL) b
*Repandae* (2n = 4x = 48)	*Nicotiana nudicaulis*	964750051	nud1	Nijmegen Botanical Garden (NL) b
	*Nicotiana nudicaulis*	A14750212	nud2	Nijmegen Botanical Garden (NL) b
	*Nicotiana nudicaulis*	A14750211	nud3	Nijmegen Botanical Garden (NL) b
	*Nicotiana nudicaulis*	964750114	nud4	Nijmegen Botanical Garden (NL) b
	*Nicotiana nudicaulis*	TW90	nud5	USDA (US) d
	*Nicotiana repanda*	994750061	rep1	Nijmegen Botanical Garden (NL) b
	*Nicotiana repanda*	994750063	rep2	Nijmegen Botanical Garden (NL) b
	*Nicotiana repanda*	994750064	rep3	Nijmegen Botanical Garden (NL) b
	*Nicotiana repanda*	994750067	rep4	Nijmegen Botanical Garden (NL) b
	*Nicotiana repanda*	994750068	rep5	Nijmegen Botanical Garden (NL) b
	*Nicotiana repanda*	TW110	rep6	USDA (US) d
	*Nicotiana nesophila*	ITB609	isl1	Tobacco Institute of Bergerac (F) e,f
	*Nicotiana nesophila*	TW87	isl2	USDA (US) d
	*Nicotiana stocktonii*	974750101	isl3	Nijmegen Botanical Garden (NL) b
	*Nicotiana stocktonii*	TW126	isl4	USDA (US) d

a labeled as *Nicotiana palmeri*, a name now considered a synonym of *N. obtusifolia.*

b http://www.bgard.science.ru.nl/.

c accession kindly provided by Dr P. Maliga, Rutgers University, NJ, USA.

d http://www.ars-grin.gov/.

e http://www.imperial-tobacco-bergerac.com/.

f direct donations from T. H. Goodspeed [60].

*Nicotiana nesophila* and *N. stocktonii* are morphologically similar and genetically closely related ([37]; also see below). As they likely represent taxonomic groups rather than biological species (S. Knapp, NHM London, personal communication), we pooled them as the Revillagigedo Islands (the oceanic Mexican islands where they occur naturally) taxa. Therefore, the statistical treatment of SSAP fragments was conduced within and among five taxa with nearly balanced sampling size: (i) *N. obtusifolia* (section *Trigonophyllae*; tri1-tri6), (ii) *N. sylvestris* (section *Sylvestres*; syl1-syl4), (iii) *N. nudicaulis* (nud1-nud5), (iv) *N. repanda* (rep1-rep6) and (v) Revillagigedo-Islands species (incl. *N. nesophila* and *N. stocktonii*; isl1-isl4).

### Sequence-specific Amplification Polymorphism (SSAP)

The SSAP technique [45] was applied as described in [46], using the ^33^P-labelled TE primers described in Table 2. The procedure was repeated on each individuals to ensure reproducibility of profiles. Reproducible bands were manually scored as present (1) or absent (0) in each accession and recorded in a data matrix for each TE.

**Table 2:** Transposable elements investigated in this study.

Name	Type a	Locus	SSAP primer (name and sequence 5′-3′)	Reference
Au	SINE	U35619	Au-1F: AAG GCT GCG TAC AAT AGA CCC	[43]
TS	SINE	D17453	TS-a: CTC CCC ACC TTG CTC TTG	[42]
Ns1	MITE	X14059	Ns1-1F: TCG TGC TCA GTC AAA CAG GTT C	[44]
Nt2	MITE	X51599	Nt2-1F: AAC TCC GTG TCG AGT CAA AC	[44]
Tnt1	LTR	X13777	Tnt1-Ol16: TTC CCA CCT CAC TAC AAT ATC GC	[39]
Tnt2	LTR	EF437960	Tnt2d: CCG AAC CTC GTA AAT TCT GGT G	[36]
TRIM	LTR	AF231351	TRIM-b: CCC GAA AGA GCC GAT GTG	[40]

a SINE for short interspersed nuclear elements; MITE for miniature inverted-repeat transposable elements; LTR for long terminal repeat retrotransposons.

### Phylogenetic Reconstruction

Relationships among *Nicotiana* accessions assessed with SSAP were evaluated by the neighbor-net method using SplitsTree 4.10 [47], [48]. The neighbor-net diagram was produced from distances computed as the proportion of positions at which two binary sequences differ (UncorrectedP option). Bootstrap support was estimated with 1000 replicates, and a single diagram representing a 95% confidence set for the networks was estimated. This procedure was applied to each TE dataset independently.

### Genetic Diversity for the Different TEs

Allele frequencies of SSAP bands were computed through the Bayesian method of Zhivotovsky [49] using non-uniform prior distributions of allele frequencies with *F*_IS_ = 1 in AFLP-surv [50], [51]. The frequency of recessive alleles, estimated by taking the sample size into account, was used to calculate the proportion of polypmorphic loci at the 5% level and Nei’s gene diversity for each taxon as well as each TE.

### Distribution of the SSAP Fragments within and Among Taxa

For each TE, SSAP bands specific to *N. sylvestris* and *N. obtusifolia* and those shared by both were counted. Whether the proportion of specific SSAP bands was significantly different in the two taxa was assessed by Yate’s one-sided Chi-square test, using SPSS 16.0.

SSAP profiles from *N. sylvestris* and *N. obtusifolia* were summed, and bands in the *Repandae* taxa showing deviation from parental additivity were counted for each allopolyploid taxon and each TE. All proportions were presented with 95% confidence intervals, following [52]. Differences in proportions were tested by multiple Yate’s one-sided Chi-square tests (or Fisher exact tests where appropriate), using SPSS 16.0, and significance was assessed at α = 0.05 with sequential Bonferroni correction for multiple comparisons [53].

Multiple Yate’s one-sided Chi-square tests with sequential Bonferroni correction (α = 0.05) were applied to the proportion of deviating SSAP bands and the ratio between new vs. missing SSAP bands, representing the relative importance of gain and loss. The distribution of deviating SSAP bands was also recorded as bands specific to each polyploid taxon or shared by two or three polyploid taxa. The total numbers of deviating SSAP bands were compared with one-way ANOVA and *post-hoc* pair-wise comparisons based on Tukey's honestly significant difference test. Finally, the parental origin of the SSAP bands lost in the polyploid taxa was recorded for each TE and deviation from the 1∶1 ratio was tested by Yate’s one-sided Chi-square test, using SPSS 16.0.

## Results

### Phylogenetic Analysis of TE Insertion Polymorphisms in Section *Repandae*

**Figure 1:**
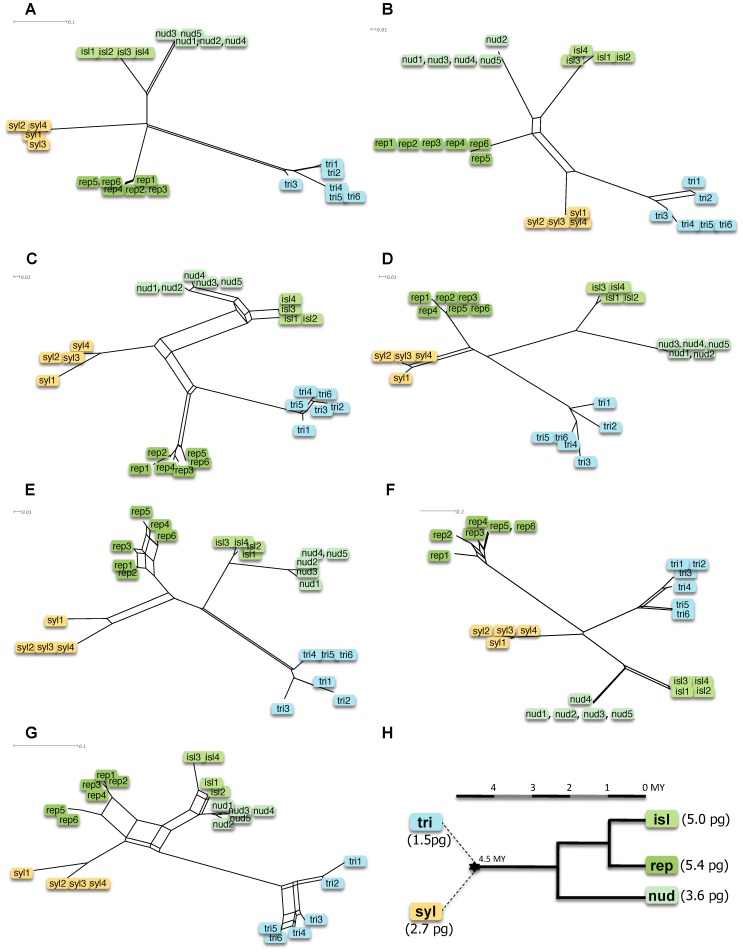
Phylogenetic reconstruction of *Nicotiana* section *Repandae.* 95% confidence neighbor-net diagrams based on SSAP profiles: (a) Au, (b) TS, (c) Ns1, (d) Nt2, (e) Tnt1, (f) Tnt2 and (g) TRIM. (h) Calibrated species tree redrawn from [32], [37], with genome sizes taken form [38]. TRI = diploid *N. obtusifolia* representing the paternal progenitor; SYL = diploid *N. sylvestris* representing the maternal progenitor; NUD = allopolyploid *N. nudicaulis*; REP = allopolyploid *N. repanda*; ISL = allopolyploids from the Revillagigedo-Islands (*N. nesophila* and *N. stocktonii*). See Table 1 for details on the 25 accessions.

SSAP gave a total of 594 reproducible bands: 108 with Au, 62 with TS, 89 with Ns1, 86 with Nt2, 80 with Tnt1, 97 with Tnt2 and 72 with TRIM (Table S1 and Figure S1). The neighbor-net for each TE resolved all accessions into taxonomic species (Figure 1). *Nicotiana sylvestris* (syl) and *N. obtusifolia* (tri) accessions formed two well-supported clusters. Accessions from section *Repandae* also formed coherent species groups (i.e. *N. nudicaulis* (nud), *N repanda* (rep), *N. nesophila* and *N. stocktonii*). The last two taxonomic species were close to each other and formed a Revillagigedo-Islands cluster (isl).

Most neighbor-nets results supported the hybrid origin of the *Repandae* clade since they were placed at the intersection of the splits connecting them to the descendents of the parental progenitors. *Nicotiana nudicaulis*, *N. nesophila* and *N. stocktonii* were usually clustered, whereas *N. repanda* formed a more distant group. Star-like topologies, such as revealed for Au and Tnt2, indicated that the allopolyploid lineages share similarities with both parents, but also that *N. repanda* is differentiated from the other polyploids. Topologies for Nt2, Tnt1 and TRIM placed *N. repanda* closer to *N. sylvestris*, whereas Ns1 placed *N. repanda* closer to *N. obtusifolia*, but are in agreement with a hybrid origin of *N. repanda*. In marked contrast, the topology for TS clustered all allopolyploid species into one group, highlighting a particular dynamic of TS as compared to the other TEs in the polyploid genomes.

Patterns of differentiation for Au and TS were not significantly associated with those of other TEs (Table S2). Au and TS each had specific distributions among taxa and the two SINEs were distinct from the other TEs. In contrast, MITEs and LTR retrotransposons produced similar patterns of differentiation.

### Genetic Diversity and Distribution of TE Insertions in the Diploid Progenitor Taxa

**Figure 2:**
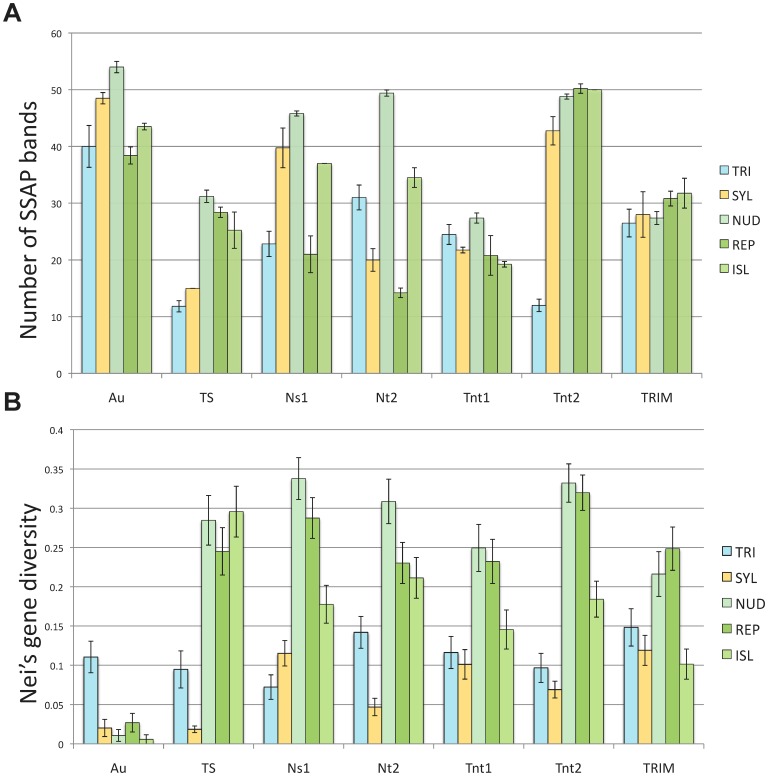
Genetic diversity and distribution of TE insertions in *Nicotiana* section *Repandae*. (a) Number of SSAP bands and (b) Nei’s gene diversity for each TE within all *Nicotiana* taxa related to the allopolyploid section *Repandae*. Error bars represent the standard error. TRI = diploid *N. obtusifolia* representing the paternal progenitor; SYL = diploid *N. sylvestris* representing the maternal progenitor; NUD = allopolyploid *N. nudicaulis*; REP = allopolyploid *N. repanda*; ISL = allopolyploids from the Revillagigedo-Islands (*N. nesophila* and *N. stocktonii*).

Transposable elements showed significant differences in number of SSAP bands shared by both diploid taxa (Table 3). Low frequencies of shared bands (<8%) were observed for TS, Tnt1 and Tnt2, higher frequencies (>16%) for TRIM and Au, and intermediate levels for Ns1 and Nt2. The number of SSAP bands and Nei’s gene diversity (Figure 2) showed contrasting tendencies and suggested different genetic loads for most TEs among diploid taxa.

**Table 3:** Distribution of SSAP bands from the different TEs within and among the diploid taxa (TRI for *Nicotiana obtusifolia* and SYL for *N. sylvestris*).

	Total	TRI/SYL a	TRI-sp a	SYL-sp a	TRI-sp *vs*. SYL-sp (Chi-Square) b
Au	87	15 (17.2%)	37 (42.5%)	35 (40.2%)	NS
TS	30	2 (6.7%)	15 (50.0%)	13 (43.3%)	NS
Ns1	66	10 (15.1%)	18 (27.3%)	38 (57.6%)	T<S: 12.89 ***
Nt2	58	8 (13.8%)	35 (60.3%)	15 (25.9%)	T>S: 14.44 ***
Tnt1	56	3 (5.3%)	31 (55.4%)	22 (39.3%)	NS
Tnt2	63	5 (7.9%)	19 (30.2%)	39 (61.9%)	T<S: 12.45 ***
TRIM	66	11 (16.6%)	30 (45.5%)	25 (37.9%)	NS
Total	426	54	185	187	

a TRI/SYL = all SSAP bands that were shared by at least one accession in both taxa; TRI-sp and SYL-sp = SSAP bands that are restricted to accessions of *N. obtusifolia* and *N. sylvestris*, respectively.

b Comparison of SSAP bands proportions that are specific to *N. obtusifolia* or *N. sylvestris* as regards to the total number of bands observed within taxa (Yate’s one-sided chi-square tests). NS: non-significant; T<S: significantly higher proportion of SSAP bands specific to *N. sylvestris* as compared to *N. obtusifolia*-specific bands; T>S: significantly higher proportion of SSAP bands specific to *N. obtusifolia* as compared to *N. sylvestris*-specific bands;

*** : significant at α = 0.001.

For Au and TS, the number of bands was significantly lower in *N. obtusifolia* than *N. sylvestris*, whereas Nei’s gene diversity was significantly higher, indicating that SINE insertions are numerous, but mostly fixed in *N. sylvestris*. The number of Ns1 bands and Nei’s gene diversity were significantly lower in *N. obtusifolia* than in *N*. *sylvestris*, whereas the opposite was observed for Nt2. This indicates that when MITE insertions are numerous in a diploid species, they are not fixed and segregate in the population. For LTR retrotransposons, the number of bands was significantly different between diploid progenitors, except for TRIM. Tnt1 showed more bands within *N. obtusifolia*, whereas Tnt2 showed more within *N. sylvestris*, but Nei’s gene diversity was not significantly different.

### Genetic Diversity in the Allopolyploid Taxa and Deviation from Expected Profiles

The number of SSAP bands and genetic diversity within allopolyploid taxa was variable for all TEs (Figure 2). In particular, *N. nudicaulis* and *N. repanda* revealed similar levels of Nei’s gene diversity for most TEs, except Nt2. Genetic diversity was significantly lower within the Revillagigedo-Islands species. Notable exceptions to this otherwise consistent pattern were observed with the SINEs. Au showed numerous bands but low gene diversity in all allopolyploids. In contrast, TS revealed significantly more bands and higher gene diversity in polyploids compared to diploids. In particular, TS revealed significantly higher diversity than other TEs in the Revillagigedo-Islands species.

For all TEs, SSAP profiles in the allopolyploid taxa strongly deviated from expected additivity (Table 4), which was roughly similar for all TEs in each polyploid taxon (average: 85.1%) with few significant differences: only Au in *N. nudicaulis* had a significantly lower proportion of non-additive bands than TS and Tnt2 (Fisher exact test; p = 0.0015 and p = 0.0093, respectively).

**Table 4:** Proportions of SSAP bands showing deviation from the expected additivity of diploid profiles in the allopolyploid *Nicotiana* section *Repandae* [NUD = *N. nudicaulis*; REP = *N. repanda*; ISL = Revillagigedo-Islands taxon (*N. nesophila* and *N. stocktonii*)].

	Au^a^	TS^a^	Ns1^a^	Nt2^a^	Tnt1^a^	Tnt2^a^	TRIM^a^
NUD	0.733 [0.628–0.819]	0.953 [0.830–0.992]	0.855 [0.737–0.927]	0.767 [0.651–0.855]	0.851 [0.738–0.922]	0.900 [0.788–0.959]	0.797 [0.668–0.886]
REP	0.840 [0.738–0.909]	0.927 [0.790–0.981]	0.912 [0.822–0.977]	0.867 [0.749–0.937]	0.875 [0.753–0.944]	0.864 [0.745–0.936]	0.774 [0.635–0.873]
ISL	0.817 [0.713–0.891]	0.891 [0.756–0.959]	0.903 [0.795–0.960]	0.809 [0.692–0.890]	0.844 [0.727–0.919]	0.912 [0.800–0.967]	0.769 [0.628–0.870]

a 95% confidence interval between brackets.

### Distribution of New SSAP Bands in Allopolyploids

SSAP detected up to 170 bands in allopolyploid accessions that were not present in any of the diploids, representing a 39.9% increase in the expected number of bands relative to the progenitors (Table 5). TS and Tnt2 showed a significantly higher proportion of new bands than other TEs, except Nt2, whereas TRIM showed significantly lower proportion of new bands compared to TS and Tnt2.

**Table 5:** Distribution of non-additive SSAP bands for TEs in allopolyploid *Nicotiana* section *Repandae* [NUD = *N. nudicaulis*; REP = *N. repanda*; ISL = Revillagigedo-Islands taxa (*N. nesophila* and *N. stocktonii*)].

	In all *Repandae* a	In NUD b	In REP b	In ISL b	Shared by NUD, REP and ISL b	Shared by NUD and REP b	Shared by NUD and ISL b	Shared by REP and ISL b	Specific to NUD b	Specific to REP b	Specific to ISL b
New bands in allopolyploids											
Au	21 (24.1) ^l,m^	17 (81) ^l^	11 (52.4) ^l^	12 (57.1) ^l^	3 (14.3)	6 (28.6)	7 (33.3)	0 (0)	1 (4.8)	2 (9.5)	2 (9.5)
TS	32 (106) ^n^	22 (68.8) ^l^	19 (59.4) ^l^	21 (65.6) ^l^	8 (25)	4 (12.5)	6 (18.8)	4 (12.5)	4 (12.5)	3 (9.4)	3 (9.4)
Ns1	23 (34.8) ^l,m^	17 (73.9) ^l^	9 (39.1) ^l,m^	14 (60.9) ^l^	3 (13)	0 (0)	10 (43.5)	1 (4.3)	4 (17.4)	5 (21.7)	0 (0)
Nt2	28 (48.3) ^l,m,n^	24 (85.7) ^l^	5 (17.9) ^m^	17 (60.7) ^l^	2 (7.1)	2 (7.1)	12 (42.9)	0 (0)	8 (28.6)	1 (3.6)	3 (10.7)
Tnt1	21 (37.5) ^l,m^	14 (66.7) ^l,m^	7 (33.3) ^l,m^	8 (38.1) ^l^	1 (4.8)	0 (0)	6 (28.6)	0 (0)	7 (33.3)	6 (28.6)	1 (4.8)
Tnt2	34 (54.0) ^l,n^	20 (58.8) ^m^	24 (70.6) ^l^	20 (58.8) ^l^	9 (26.5)	4 (11.8)	6 (17.6)	2 (5.9)	1 (2.9)	9 (26.5)	3 (8.8)
TRIM	11 (16.7) ^m^	8 (72.7) ^l^	9 (81.8) ^l^	7 (63.6) ^l^	4 (36.4)	2 (18.2)	2 (18.2)	1 (9.1)	0 (0)	2 (18.2)	0 (0)
All TEs c	170 (39.9)	122 (71.8)	82 (48.2)	99 (58.2)	30 (17.6) ^l,m^	18 (10.6) ^l^	49 (28.8) ^m^	8 (4.7) ^l^	25 (14.7) ^l,m^	28 (16.5) ^l,m^	12 (7.1) ^l,m^
Parental bands lost in allopolyploids											
Au	71 (81.6) ^L^	49 (56.3) ^L^	57 (65.5) ^L,M^	55 (63.2) ^L^	35 (49.3)	3 (4.2)	8 (11.3)	9 (12.7)	3 (4.2)	10 (14.1)	3 (4.2)
TS	26 (86.7) ^L^	19 (63.3) ^L,M^	19 (63.3) ^L,M^	23 (76.7) ^L,M^	14 (53.8)	2 (7.7)	2 (7.7)	3 (11.5)	1 (3.8)	0 (0)	4 (15.4)
Ns1	58 (87.9) ^L^	36 (54.5) ^L^	43 (65.2) ^L,M^	42 (63.6) ^L,M,N^	24 (41.4)	1 (1.7)	9 (15.5)	5 (8.6)	2 (3.4)	13 (22.4)	4 (6.9)
Nt2	51 (87.9) ^L^	32 (55.2) ^L^	46 (79.3) ^M^	38 (65.5) ^L,M,N^	28 (54.9)	0 (0)	3 (5.9)	6 (11.8)	1 (2)	12 (23.5)	1 (2)
Tnt1	54 (96.4) ^L^	43 (76.8) ^M^	42 (75) ^M^	46 (82.1) ^M^	31 (57.4)	0 (0)	10 (18.6)	5 (9.3)	2 (3.7)	6 (11.1)	0 (0)
Tnt2	45 (71.4) ^M^	34 (54) ^L^	27 (42.9) ^L^	32 (50.8) ^N^	14 (31.1)	2 (4.4)	13 (28.9)	5 (11.1)	5 (11.1)	6 (13.3)	0 (0)
TRIM	46 (69.7) ^M^	39 (59.1) ^L,M^	32 (48.5) ^L^	33 (50) ^N^	24 (52.2)	2 (4.3)	7 (15.2)	1 (2.2)	6 (13.0)	5 (10.9)	1 (2.2)
All TEs c	351 (82.4)	252 (59.2)	266 (62.4)	269 (63.1)	170 (48.4) ^L^	10 (2.8) ^M^	52 (14.8) ^N^	34 (9.7) ^M,N^	20 (5.7) ^M,N^	52 (14.8) ^N^	13 (3.4) ^M,N^

a calculated as number (percentage) of new or missing bands relative to parental additivity. Lines not sharing a common letter (l, m or n; L, M or N) are significantly different as assessed by multiple Chi-Square tests.

b calculated as number (percentage) of new or missing bands relative to corresponding bands in indicated *Repandae* species. Lines not sharing a common letter (l, m or n; L, M or N) are significantly different as assessed by multiple Chi-Square tests.

c rows not sharing a common letter (l, m or n; L, M or N) are significantly different as assessed by one-way ANOVA with post-hoc Tukey tests on pair-wise comparisons.

New SSAP bands were highly similar among polyploid lineages (Table 5) and unevenly distributed among groups of taxa (Table 6). Comparisons involving *N. repanda* showed a low proportion of shared new bands, especially for Ns1 and Nt2. Less than half the total new SSAP bands were restricted to only one polyploid taxon, with a maximum of 16.5% specific to *N. repanda*, 14.7% specific to *N. nudicaulis* and 7.1% specific to the Revillagigedo-Islands species; 26.5% of new Tnt2 bands were specific to *N. repanda*.

**Table 6:** One-way ANOVA on the distribution of new and missing SSAP bands among the different groups of polyploid lineages in *Nicotiana* section *Repandae.*

	Sum of Squares	df	Mean of Squares	F	p-value
New SSAP bands					
Between groups	0.2855	6	0.0476	4.981	0.0006
Within groups	0.4005	42	0.0096		
Lost SSAP bands					
Between groups	1.0436	6	0.1739	44.100	<0.0001
Within groups	0.1656	42	0.0038		

### Distribution and Origin of Lost SSAP Bands in the Allopolyploids

**Figure 3:**
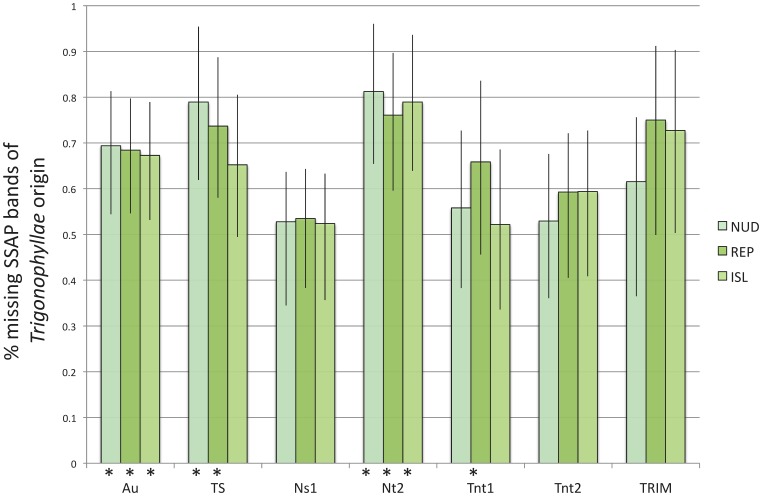
Origin of missing SSAP bands in the allopolyploid ***Nicotiana*** section *Repandae.* Missing bands are represented as the percentage of bands of paternal origin. * indicates when missing SSAP bands of *N. obtusifolia* origin are significantly more frequent than bands of *N. sylvestris*. Error bars represent the 95% confidence intervals. NUD = *N. nudicaulis*; REP: *N. repanda*; ISL: Revillagigedo-Islands taxa (*N. nesophila* and *N. stocktonii*).

Among the 426 SSAP bands present in diploids, 351 bands (82.4%) were not detected in one or more allopolyploid taxa (Table 5). Most TEs had similar levels of missing bands. Most SSAP bands missing among polyploid lineages (Table 5) were unevenly distributed among groups (Table 6). Few missing SSAP bands were restricted to only one polyploid taxon (14.8% in *N. repanda*, 5.7% in *N. nudicaulis* and 3.4% in the Revillagigedo-Islands species). Band absence was comparatively important in *N. repanda* for Ns1 (22.4%) and Nt2 (23.5%). SSAP bands that were missing in the polyploid taxa were mostly of *N. obtusifolia* origin (Figure 3). This was particularly true in all polyploids for Au and Nt2, in *N. nudicaulis* and *N. repanda* for TS, in *N. repanda* and Revillagigedo-Islands species for TRIM, and in *N. repanda* for Tnt1.

For most TEs, the proportion of missing SSAP bands was significantly higher than that for new bands. As exceptions, new bands were significantly more frequent for TS in all taxa, Nt2 in *N. nudicaulis* and Tnt2 in *N. repanda* (Table 5).

## Discussion

### Evolutionary Dynamics of *Repandae* Genomes

**Figure 4:**
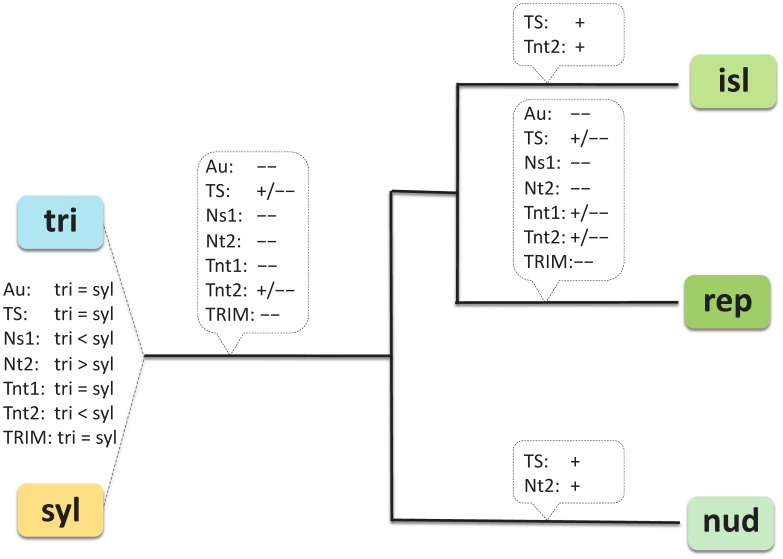
Evolutionary dynamics of TEs in the allopolyploid *Nicotiana* section *Repandae*. Relative abundance of the various TE families in the progenitor species (TRI = diploid *N. obtusifolia* representing the paternal progenitor; SYL = diploid *N. sylvestris* representing the maternal progenitor) and evidence of sequence amplification (+) and loss (–) along the evolutionary path leading to the polyploid species (NUD = *N. nudicaulis*; REP = *N. repanda*; ISL = Revillagigedo-Islands taxa, *N. nesophila* and *N. stocktonii*).

For extant descendents of the progenitors of *N.* section *Repandae* as well as polyploid taxa, phylogenetic relationships among have been unambiguously identified [32], [37]. Phylogenetic networks reconstructed with SSAP supported the hybrid origin of section *Repandae*. In contrast to analyses based on morphology and molecular analyses that placed *N. nudicaulis* as sister to the other polyploids [32], [37], SSAP markers grouped *N. nudicaulis* and the Revillagigedo-Islands taxa, whereas *N. repanda* consistently fell as a more distant group. This indicates that the TE fraction of *N. repanda* evolved with a unique trajectory. *Nicotiana repanda* indeed exhibited losses for all TEs and specific amplification of Tnt1, Tnt2 and TS (Figure 4). Furthermore, missing bands in *N. repanda* were mostly of *N. obtusifolia* origin for Au, Nt2 and Tnt1, and of *N. sylvestris* origin for Ns1, explaining why *N. repanda* was more closely associated with the maternal or the paternal progenitors, respectively. Phylogenetic trees derived from TE insertion patterns thus reveal specific evolutionary dynamics of different TE fractions, which may be loosely similar to evolutionary relationships among species [54].

Molecular cytogenetics revealed genomic restructuring of *Nicotiana* genomes in less than 5 million years [34]. The present analysis highlights considerable restructuring of TE fractions in *Repandae* polyploids. SSAP addresses TE dynamics at a genome-wide scale [54], but also reveals molecular changes at insertion sites, such as those highlighted with AFLP [13]. Statistically relevant interpretations of the evolutionary dynamics of TEs using SSAP should thus include as much biological diversity as possible and require a large number of loci. Our interpretations are based on conservative assumptions and focused on comparative patterns among several categories of TE. Frequencies of missing bands observed in *Repandae* allopolyploids were fairly constant for most TEs (from 69.7% to 96.4%), but there was a six-fold variation in new SSAP bands depending on which TE is being considered (from 16.7% to 106%), indicating that new SSAP bands are novel insertion events to a certain extant.

In addition, we cannot be certain of which aspects of TE diversity in allopolyploids occurred during allopolyploid diversification or preexisted at the diploid level. However, 17.6% of the new SSAP bands were shared by all polyploid lineages, and over 25% for TS and Tnt2, which suggests these new bands appeared soon after polyploidization, and before divergence of extant *Repandae* species. As Tnt2 and TS show other evidence for recent activity [36], [41], a burst of amplification was possibly associated with genomic shock of allopolyploidy. Similarly, 48.4% of the missing bands were absent from all polyploids, suggesting that restructuring around TE insertions occurred in the ancestral polyploid taxon. For most TEs, polyploid taxa chiefly lost SSAP bands of *N. obtusifolia* origin, indicating predominant restructuring of paternal TE genome fractions in polyploids. We cannot exclude the possibility that parental polymorphic insertions have disappeared in descendants of the diploid progenitors.

### Contrasting Evolutionary Dynamics of the Different TEs

Few SSAP bands were shared by both diploid taxa. As expected, TS and Tnt2 revealed low frequencies of shared bands, whereas ancient TEs such as TRIM and Au [40], [41] showed higher frequencies of shared bands. Most TEs showed different levels of diversity in the two diploids, suggesting considerable divergence of TE genome fractions since the diversification of diploid taxa.

SINEs had comparable patterns of diversity in the diploids, but showed contrasting dynamics at the polyploid level. A low ratio of new/missing bands suggests deletion leading to band loss as the dominant process for Au during diploidization, whereas a relatively high ratio of new/missing SSAP bands in the polyploids suggests TS transpositional activity during diploidization. Accordingly, TS recently proliferated in the *N. tabacum* lineage, whereas Au is less conserved [41].

MITEs showed differential dynamics in the diploid progenitors, with Ns1 having been more active in *N. sylvestris* and Nt2 more active in *N. obtusifolia*, both exhibiting a relatively high genetic diversity in polyploids suggestive of TE fraction restructuring after polyploidization. In particular, both MITEs showed a high new/missing ratio in *N. nudicaulis* and a low new/missing ratio in *N. repanda*. This suggests that these two host genomes have imposed contrasting constraints on the restructuring of MITE fractions.

For LTR retrotransposons, most deviating SSAP bands were shared by all polyploid taxa, suggesting that restructuring in the corresponding TE fractions occurred in the single ancestral polyploid. Tnt2 had a high ratio of new/missing SSAP bands suggesting amplification in the polyploids, in accordance with observations in the young allopolyploid *N. tabacum* [36]. The ratio of new/missing SSAP bands was, however, lower in *N. nudicaulis* than in the other polyploids, indicating specific transposition of Tnt2 in the ancestor of *N. repanda* and the island taxa. TRIM showed the exact opposite pattern, with a relatively low ratio of new/missing SSAP bands, suggesting shrinkage of the corresponding TE fraction in the polyploids. This pattern was similar to Au and correlates with the high sequence heterogeneity observed in *N. tabacum* for these two ancient TE populations (Deloger and Grandbastien,unpublished data, [41]). A significantly higher ratio in *N. nudicaulis* also suggests specific restructuring of TRIM insertions in the common ancestor of *N. repanda* and the island taxa. An intermediate situation is observed for Tnt1, a retrotransposon population displaying lower activity levels than Tnt2 in *N*. *tabacum* [36].

Our survey of *N.* section *Repandae* provides evidence of differential restructuring of each TE population in polyploids, as well as between each allopolyploid species (summarized in Figure 4). Possible mechanisms underlying such differential dynamics are still unknown. The differential long-term dynamics of TEs in allopolyploid section *Repandae* does not seem to be primarily dependent on TE type, as the TEs investigated here typically showed more contrasting dynamics within than among TE categories (e.g. SINEs, MITEs, LTR retrotransposons). Furthermore, TE populations selected here based on their differential dynamics in *N. tabacum* displayed comparable dynamics in the *Repandea* lineages, suggesting that their dynamics is explained by intrinsic factors. Noticeably, contrasting dynamics matched closely the TE relative abundance in diploids, which supports the hypothesis that the accumulation of divergent TE loads in progenitors determines strength of the initial genome shock and TE dynamics after hybridization [14], [18], [23], [55].

### Transposable Elements and Long-term Diploidization

Our results suggest that genome TE-associated restructuring likely played a major role during the long-term diploidization of *N.* section *Repandae* and was driven by differential dynamics of TEs [14], [56]. Although dynamics of TEs have a dramatic impact on genome size [57], the specific dynamics of TE genome fraction assessed here loosely fit changes in genome size among species of section *Repandae*. The proliferation of the LTR retrotransposon Tnt2 in *N. repanda* and the Revillagigedo-Islands taxa might explain part of the genome size increase in these taxa (28.6% and 19.1%, respectively; [38]), but does not exclude the role of other repetitive sequences [58]. Indeed, in a recent analysis of genomic DNA using next generation sequencing technologies it has been shown that much of the genomic expansion observed in *N. repanda* is accounted for by chromovirus-like Ty3 gypsy elements (Renny Byfield et al. unpublished data).

Evolutionary forces underlying observed genomic dynamics during diploidization in allopolyploid section *Repandae* still remain unclear. Noticeably, our results revealed significantly lower genetic diversity in the Revillagigedo-Islands taxa for most TEs. It is tempting to speculate that this reduced diversity results from demographic factors such as bottlenecks during and after the island colonization [59]. To what extent different evolutionary forces other than genetic drift have shaped contrasting TE arrangements in larger populations of *N. nudicaulis* and *N. repanda* remains to be explored. This survey suggests that demographic features of host taxa imposes evolutionary constraints and, together with intrinsic features of TEs themselves, may have a significant impact on the evolution of TE fractions [56]. Future studies addressing the fate of TE insertions and genome evolution under the influence of TEs should emphasize evolutionary processes acting at the level of the host and sample naturally occurring populations throughout their ranges.

## Supporting Information

Figure S1 **Example of SSAP profile obtained for TRIM obtained on accessions of** ***Nicotiana*** **section** ***Repandae.***
(DOC)

Table S1 **Number of SSAP bands in each accession of** ***Nicotiana*** **section** ***Repandae*** **and measures of genetic diversity.**
(DOC)

Table S2 **Multiple Mantel tests for each TE.**
(DOC)
