# Supplementary material for: Differential Dynamics of Transposable Elements during Long-Term Diploidization of Nicotiana Section Repandae (Solanaceae) Allopolyploid Genomes
Source: PLoS One. 2012 Nov 21;7(11):e50352. doi: 10.1371/journal.pone.0050352 (PMC3503968; doi:10.1371/journal.pone.0050352)
Supplement: Figure S1 — Example of SSAP profile obtained for TRIM obtained on accessions of Nicotiana section Repandae. (DOC) [file pone.0050352.s001.doc]

**Figure S1:** Example of SSAP profile obtained for TRIM obtained on accessions of *Nicotiana* section *Repandae* (see Table 1 for further information on the accessions). TRI = section *Trigonophyllae* (descendants of the diploid paternal progenitor); NESO = N. nesophila; NUDI = N. nudicaulis; REP = N. rependa; STO = N. stocktonii; SYLV = section *Sylvestres* (descendant of the maternal progenitor); * internal reference
