# Supplementary material for: Differential Dynamics of Transposable Elements during Long-Term Diploidization of Nicotiana Section Repandae (Solanaceae) Allopolyploid Genomes
Source: PLoS One. 2012 Nov 21;7(11):e50352. doi: 10.1371/journal.pone.0050352 (PMC3503968; doi:10.1371/journal.pone.0050352)
Supplement: Table S1 — Number of SSAP bands in each accession of Nicotiana section Repandae and measures of genetic diversity. (DOC) [file pone.0050352.s002.doc]

**Table S1:** Number of SSAP bands in each accession of *Nicotiana* section *Repandae* and measures of genetic diversity (% polymorphic loci = PLP; Nei’s gene diversity = Nei; Shannon diversity index = H) within the taxa used in this study.

| Abbreviation | Au | TS | Ns1 | Nt2 | Tnt1 | Tnt2 | TRIM |
| --- | --- | --- | --- | --- | --- | --- | --- |
| syl1 | 48 | 15 | 45 | 23 | 21 | 39 | 34 |
| syl2 | 48 | 15 | 38 | 19 | 22 | 44 | 26 |
| syl3 | 50 | 15 | 38 | 19 | 22 | 44 | 26 |
| syl4 | 48 | 15 | 38 | 19 | 22 | 44 | 26 |
| PLP | 3.7 | 0 | 53.9 | 26.7 | 35 | 45.4 | 50 |
| Nei | 0.020 | 0.019 | 0.115 | 0.047 | 0.101 | 0.069 | 0.119 |
| H | 0.015 | 0 | 0.067 | 0.023 | 0.065 | 0.016 | 0.078 |
| tri1 | 39 | 12 | 19 | 29 | 24 | 10 | 26 |
| tri2 | 38 | 13 | 25 | 29 | 28 | 13 | 23 |
| tri3 | 34 | 10 | 23 | 35 | 24 | 12 | 30 |
| tri4 | 43 | 12 | 23 | 31 | 24 | 13 | 28 |
| tri5 | 43 | 12 | 22 | 31 | 23 | 12 | 25 |
| tri6 | 43 | 12 | 25 | 31 | 24 | 12 | 27 |
| PLP | 23.1 | 27.4 | 12.4 | 27.9 | 21.3 | 24.7 | 50 |
| Nei | 0.110 | 0.095 | 0.072 | 0.142 | 0.116 | 0.097 | 0.148 |
| H | 0.098 | 0.075 | 0.048 | 0.110 | 0.091 | 0.090 | 0.110 |
| nud1 | 55 | 31 | 46 | 50 | 28 | 49 | 26 |
| nud2 | 55 | 33 | 46 | 50 | 28 | 49 | 29 |
| nud3 | 53 | 31 | 46 | 49 | 28 | 49 | 27 |
| nud4 | 53 | 31 | 46 | 49 | 26 | 49 | 28 |
| nud5 | 54 | 30 | 45 | 49 | 27 | 48 | 27 |
| PLP | 1.9 | 69.4 | 68.5 | 62.8 | 48.8 | 76.3 | 56.9 |
| Nei | 0.011 | 0.285 | 0.338 | 0.309 | 0.249 | 0.332 | 0.216 |
| H | 0.005 | 0.045 | 0.011 | 0.029 | 0.023 | 0.010 | 0.046 |
| rep1 | 39 | 28 | 26 | 16 | 16 | 49 | 30 |
| rep2 | 37 | 28 | 23 | 15 | 16 | 49 | 30 |
| rep3 | 37 | 28 | 23 | 15 | 21 | 50 | 32 |
| rep4 | 38 | 28 | 24 | 13 | 23 | 51 | 31 |
| rep5 | 40 | 28 | 18 | 14 | 25 | 51 | 32 |
| rep6 | 40 | 30 | 17 | 14 | 19 | 50 | 29 |
| PLP | 4.6 | 61.3 | 59.6 | 50 | 48.8 | 79.4 | 65.3 |
| Nei | 0.027 | 0.245 | 0.288 | 0.230 | 0.232 | 0.320 | 0.248 |
| H | 0.009 | 0.023 | 0.014 | 0.006 | 0.030 | 0.003 | 0.026 |
| isl1 | 44 | 28 | 37 | 33 | 20 | 50 | 30 |
| isl2 | 44 | 28 | 37 | 33 | 19 | 50 | 29 |
| isl3 | 43 | 23 | 37 | 36 | 19 | 50 | 34 |
| isl4 | 43 | 22 | 37 | 36 | 19 | 50 | 34 |
| PLP | 0.9 | 69.4 | 57.3 | 61.6 | 40 | 61.9 | 45.8 |
| Nei | 0.006 | 0.296 | 0.178 | 0.211 | 0.146 | 0.184 | 0.101 |
| H | 0.023 | 0.019 | 0.083 | 0.034 | 0.071 | 0.096 | 0.089 |
