# Supplementary material for: Differential Dynamics of Transposable Elements during Long-Term Diploidization of Nicotiana Section Repandae (Solanaceae) Allopolyploid Genomes
Source: PLoS One. 2012 Nov 21;7(11):e50352. doi: 10.1371/journal.pone.0050352 (PMC3503968; doi:10.1371/journal.pone.0050352)
Supplement: Table S2 — Multiple Mantel tests for each TE. (DOC) [file pone.0050352.s003.doc]

**Table S2**: Multiple Mantel tests on Nei’s pair-wise genetic distance among taxa for each TE.

|  | Au | TS | Ns1 | Nt2 | Tnt1 | Tnt2 | TRIM |
| --- | --- | --- | --- | --- | --- | --- | --- |
| Au | - | 63.39 | 75.15 | 67.01 | 69.85 | 78.90 | 80.62 |
| TS | 0.77 | - | 72.19 | 76.92 | 58.67 | 76.92 | 79.69 |
| Ns1 | 0.87* | 0.81* | - | 92.61 | 83.62 | 90.96 | 79.03 |
| Nt2 | 0.93* | 0.74* | 1.08** | - | 84.23 | 88.11 | 81.75 |
| Tnt1 | 1.10* | 0.56 | 1.20** | 1.07** | - | 86.34 | 86.62 |
| Tnt2 | 0.90* | 0.75* | 0.95** | 0.83** | 0.75** | - | 81.75 |
| TRIM | 0.88* | 0.88* | 0.86** | 0.78** | 0.69** | 0.78** | - |

Upper diagonal of the matrix: variance explained (R^2^) for each test.

Lower diagonal of the matrix: slope of the relationship and significance after sequential Bonferonni correction at α = 0.01 (*) and 0.005 (**).

Relationships considered significant under conservative criteria (p < 0.005 and >80% of common variance) are shown in bold.
